# Effects of Heat Stress on Motion Characteristics and Metabolomic Profiles of Boar Spermatozoa

**DOI:** 10.3390/genes13091647

**Published:** 2022-09-14

**Authors:** Heming Sui, Shiqi Wang, Gang Liu, Fei Meng, Zubing Cao, Yunhai Zhang

**Affiliations:** 1 National Animal Husbandry Service, Beijing 100125, China; 2 Anhui Province Key Laboratory of Local Livestock and Poultry, Genetical Resource Conservation and Breeding, College of Animal Science and Technology, Anhui Agricultural University, Hefei 230036, China

**Keywords:** heat stress, pig, sperm, motion characteristics, metabolome

## Abstract

Heat stress (HS) commonly causes boar infertility and economic loss in the swine industry. The heat tolerance of boar semen presents obvious differences among individuals. However, whether heat stress affects motion characteristics and the metabolome profile in boar sperm remains unclear. In this study, the kinetic features of sperm from HS and non-HS (NHS) groups were detected by computer-assisted sperm analysis, and metabolomic profiling was performed by liquid chromatography–mass spectrometry. The results showed that heat stress significantly reduced sperm motility, average path distance (APD), straight-line velocity (VSL), straightness (STR), and linearity (LIN) (*p* < 0.05). A total of 528 and 194 metabolites in sperm were identified in the positive and negative ion modes, respectively. Lipids and lipid-like molecules, and organic acids and derivatives were major metabolic classes in the two modes. Furthermore, we separately identified 163 and 171 differential metabolites in the two modes between HS and NHS groups. Clustering analysis further revealed significant metabolic changes in sperm after heat stress. The Kyoto Encyclopedia of Genes and Genomes (KEGG) analysis showed that differential metabolites in the two modes were enriched in glycerophospholipid, choline, and alanine, aspartate, and glutamate and lysine metabolism. Taken together, these results demonstrate that heat stress can alter the motion characteristics and metabolomic profiles of boar sperm.

## 1. Introduction

Heat stress occurs when environmental temperatures during the summer period exceed boars’ physiological range. Heat-stressed boars commonly suffer from low reproductive performance, which leads to significant economic loss for the swine industry [1,2]. Previous studies showed that the infertility of heat-stressed boars is characterized by reduced sperm motility, concentration, and volume, as well as abnormal sperm morphology [3,4]. Spermatogenesis is highly susceptible to heat stress, but the physiological response to heat stress varies between boar individuals. It was reported that the heat tolerance of sperm presents visible differences among individuals [5]. Thus, the pre-selection of heat-tolerant boar semen could enhance utilization efficiency in artificial insemination programs. Recently, an in vitro heat stress experimental model for boar semen was established to provide a valuable tool to screen for reliable sperm biomarkers of heat stress [6].

The capacity of sperm to endure heat is recognized as a critical genetic feature in breeding [5]. Heat stress, thus, inevitably alters sperm molecular compositions at the genetic and epigenetic levels. The identification of molecular markers associated with sperm heat stress is a prerequisite to develop abatement strategies for thermal stress. Currently, multiple OMICS technologies were applied to seek heat stress markers in animal reproductive organs. At the genomic level, tropical summer could induce DNA damage in boar spermatozoa [7]. Transcriptome analysis revealed that the expression of multiple genes was altered in boars exposed to heat stress, and some differentially expressed genes were also identified between heat-tolerant and heat-susceptible boars [8]. Proteomic profiling showed that heat stress caused the differential expression of 60 and 85 proteins in human [9] and boar [10] sperm, respectively. Moreover, heat stress also severely perturbed the proteomic profiles of seminal plasma in rams [11] and Brangus bulls [12]. It was noted that subtle changes in transcriptome and proteome are eventually manifested in metabolome. Following exposure to high-temperatures, Holstein bull semen exhibited aberrant concentrations of fatty acids and cholesterol [13]. High environmental temperatures resulted in up-regulation of metabolites in rat epididymis [14]. A recent study showed that seminal plasma metabolites related to hormone secretion, energy metabolism, and fatty acid oxidation were associated with heat stress in Mediterranean buffalo bulls [15]. Although heat-stress-induced metabolic changes are clarified in some male species, whether heat stress affects metabolome in boar sperm remains unclear.

In the present study, the motion features and metabolomic profiles of boar sperm in vitro exposed to high temperatures were deeply analyzed. We found that heat stress altered the kinematic parameters of sperm and caused significant changes in metabolites associated with fatty acid and amino acid metabolism in sperm. Furthermore, differential metabolite-enriched pathways are mainly associated with sperm quality. Our results provide potential markers for screening heat-tolerant semen and developing strategies to mitigate heat stress.

## 2. Materials and Methods

### 2.1. Heat Stress Treatment for Boar Sperm

Twenty Huoshou black boars, a Chinese native pig breed, were raised under the same management conditions and fed with the same diets. The boars were healthy and had no testicular disorders. The quality of the fresh boar semen met the national criteria in China. Fresh semen from Huoshou black boars was collected using the gloved hand method. The semen was diluted in an extender at 35 °C. Following dilution, the semen was divided into two groups, in which one was incubated for 1 h at 35 °C (NHS group), and the other one was incubated for 1 h at 41 °C (HS group).

### 2.2. Evaluation of Sperm Quality

Semen from several boars was collected and pooled. The pooled semen was separated into NHS and HS groups. Semen from each group was used to analyze the kinetic parameters. One μL of semen was mixed with nine μL of diluent, and a ten-μL sample was then placed on the glass slide. The sperm motility and kinetic parameters were detected using a CASA system (IMV, AB2625S, Guangzhou, China).

### 2.3. Purification of Spermatozoa

The sperm samples were purified using a 90–45% discontinuous Percoll gradient centrifugation. The semen was carefully layered over the top of a prepared Percoll gradient fraction. The sperm samples were then pelleted by centrifugation at 950× *g* for 15 min at room temperature, and the sperm pellet was washed three times using phosphate-buffered saline.

### 2.4. Metabolite Extraction

Two-hundred μL of water was added to the samples. After 30 s of vortexing, the samples were frozen and thawed with liquid nitrogen for 3 times. The samples were then sonicated for 10 min in an ice-water bath. Fifty μL of homogenate was used to measure the protein concentration. Then, 600 μL acetonitrile: methanol = 1:1 was added to the rest part and transferred to 2 mL EP tube. Following the 30 s vortex, the samples were incubated at 40 °C for 1 h and centrifuged at 12,000 (RCF = 13,800× *g*), R = 8.6 cm) rpm for 15 min at 4 °C. Seven-hundred μL of supernatant was transferred to an EP tube and dried in a vacuum concentrator. Acetonitrile: methanol: water = 2:2:1, with isotopically-labelled internal standard mixture, was added in proportion. After 30 s of vortexing, the samples were sonicated for 10 min in ice-water bath. The samples were then centrifuged at 12,000 (RCF = 13,800× *g*), R = 8.6 cm) rpm for 15 min at 4 °C. The resulting supernatant was transferred to a fresh glass vial for analysis. The quality control (QC) sample was prepared by mixing an equal aliquot of the supernatants from all of the samples.

### 2.5. Liquid Chromatography–Mass Spectrometry (LC-MS) Analysis

LC-MS/MS analyses were performed using an UHPLC system (Vanquish, Thermo Fisher Scientific, Waltham, MA, USA) with a UPLC BEH Amide column (2.1 mm × 100 mm, 1.7 μm) coupled to a Orbitrap Exploris 120 mass spectrometer (Orbitrap MS, Thermo, Waltham, MA, USA). The mobile phase consisted of 25 mmol/L ammonium acetate and 25 ammonia hydroxide in water (pH = 9.75) (A) and acetonitrile (B). The auto-sampler temperature was 4 °C, and the injection volume was 4 μL. The Orbitrap Exploris 120 mass spectrometer was used for its ability to acquire MS/MS spectra on information-dependent acquisition (IDA) mode in the control of the acquisition software (Xcalibur, Thermo, Waltham, MA, USA). In this mode, the acquisition software continuously evaluates the full-scan MS spectrum. The ESI source conditions were set as follows: sheath gas flow rate as 50 Arb, Aux gas flow rate as 15 Arb, capillary temperature as 320 °C, full MS resolution as 60,000, MS/MS resolution as 15,000, collision energy as 10/30/60 in NCE mode, spray voltage as 3.8 kV (positive) or −3.4 Kv (negative), respectively.

### 2.6. Data Preprocessing and Annotation

The raw data were converted to the mzXML format using ProteoWizard, and processed with an in-house program, which was developed using R and based on XCMS, for peak detection, extraction, alignment, and integration. Then, an in-house MS2 database (BiotreeDB, Shanghai, China) was applied in metabolite annotation. The cutoff for annotation was set at 0.3.

### 2.7. Statistical Analysis

The results were presented as mean ± standard error of mean (mean ± S.E.M). All data were logarithmically transformed using SIMCA software (v16.2, Umea, Sweden) to normalize distributions. The independent-sample t-test and the bivariate correlation analysis were carried out using SPSS 17.0 (Armonk, New York, USA) to determine significant difference levels. Orthonormal partial least squares discriminant analysis (OPLS-DA) of metabolomic data was performed by using SIMCA-P 14.0 software (Umetrics, Umea, Sweden). Those variables with variable importance in the projection (VIP) > 1.0, *p*-value < 0.05, and fold change (FC) > 1.50 or <0.66 were identified as differentiated metabolites. Based on the differentiated metabolites, metabolic pathway analysis was performed by using MetaboAnalyst 3.0 (Baiqu, Shanghai, China). The volcano plots and heat map were generated using Origin 9.0 software (Hampton, Waltham, MA, USA).

## 3. Results

### 3.1. Effect of Elevated Ambient Temperature on Motion Characteristics in Boar Spermatozoa

**Figure 1:**
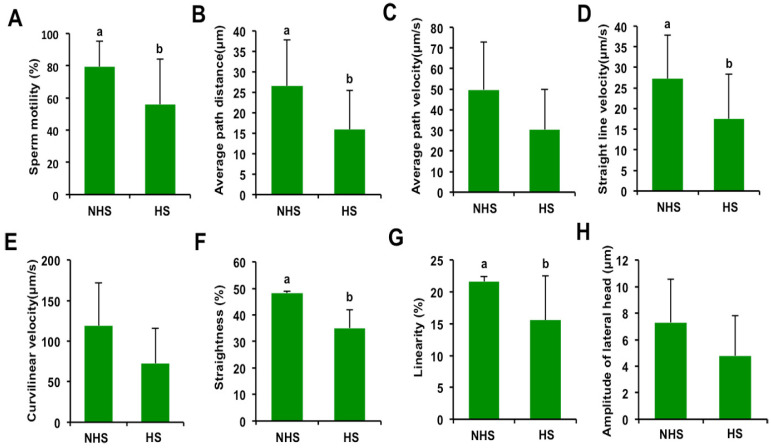
Effect of heat stress on kinetic parameters of boar sperm. (**A**) Sperm motility in NHS and HS groups. Kinetic parameters of sperm, including average path distance (**B**), average path velocity (**C**), straight line velocity (**D**), curvilinear velocity (**E**), straightness (**F**), linearity (**G**), and amplitude of lateral head (**H**), were detected in NHS and HS group. NHS: non-heat-stress, HS: heat stress. All data are shown as mean ± S.E.M, and different letters on the bars indicate significant differences (*p* < 0.05).

To determine whether high temperatures impaired boar sperm quality, kinetic parameters were analyzed using the CASA system. The results showed that HS treatment significantly reduced sperm motility (Figure 1A) (*p* < 0.05). The average path distance (APD) (Figure 1B), straight-line velocity (VSL) (Figure 1D), straightness (STR) (Figure 1F), and linearity (LIN) (Figure 1G) of sperm in the HS group were also decreased compared to the non-heat-stress (NHS) group (*p* < 0.05). However, the HS treatment did not significantly affect the following kinetic parameters: average path velocity (VAP) (Figure 1C), curvilinear velocity (VCL) (Figure 1E), and amplitude of lateral head (ALH) (Figure 1H). Therefore, these results indicate that HS impairs the kinematic features of boar sperm.

### 3.2. Changes of Metabolomics Features in Boar Spermatozoa

**Figure 2:**
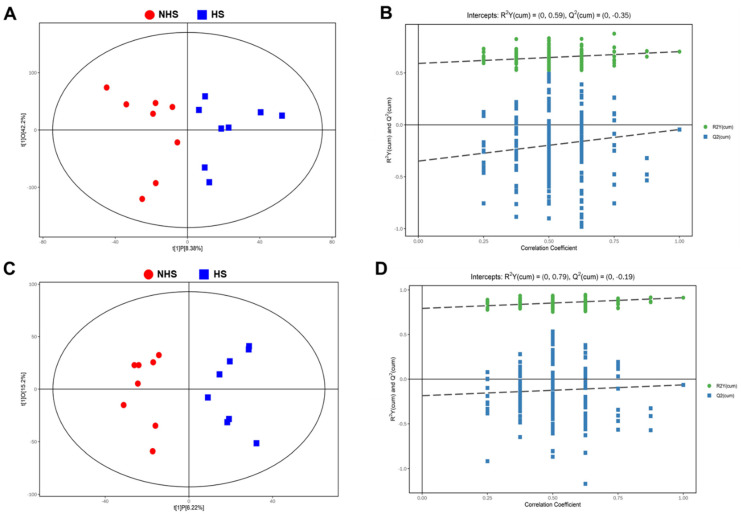
The scatter and permutation plot of OPLS-DA model. (**A**,**C**) OPLS-DA score scatter plots of sperm metabolites in the positive and negative ion modes. The plots were used to analyze the segregation of sperm samples between NHS and HS groups. Red marks NHS group, blue denotes HS group. (**B**,**D**) OPLS-DA permutation plots of sperm metabolites in the positive and negative ion modes. The slope of R2 is >0 and the Y-intercept of Q2 is <0.05, indicating a valid model.

To investigate whether heat stress altered the metabolism of boar sperm, non-targeted metabolomics sequencing was performed to identify metabolic changes. The OPLS-DA models in the positive and negative ion modes were established to compare sperm metabolites between the HS and NHS groups. As shown in Figure 2A,C, the HS and NHS groups could be clearly segregated, indicating that certain metabolic changes occurred in the sperm after heat stress. In addition, permutation tests were performed to confirm the reliability of the OPLS-DA models (Figure 2B,D). R^2^ and Q^2^ represent the interpretability and predictability of the models, respectively. R^2^ and Q^2^ in the positive and negative ion modes were near to 1 or less than zero, respectively, indicating that there was no overfitting, and the OPLS-DA models were stable and reliable.

### 3.3. Classification of Sperm Metabolites in the Positive and Negative Ion Modes

**Figure 3:**
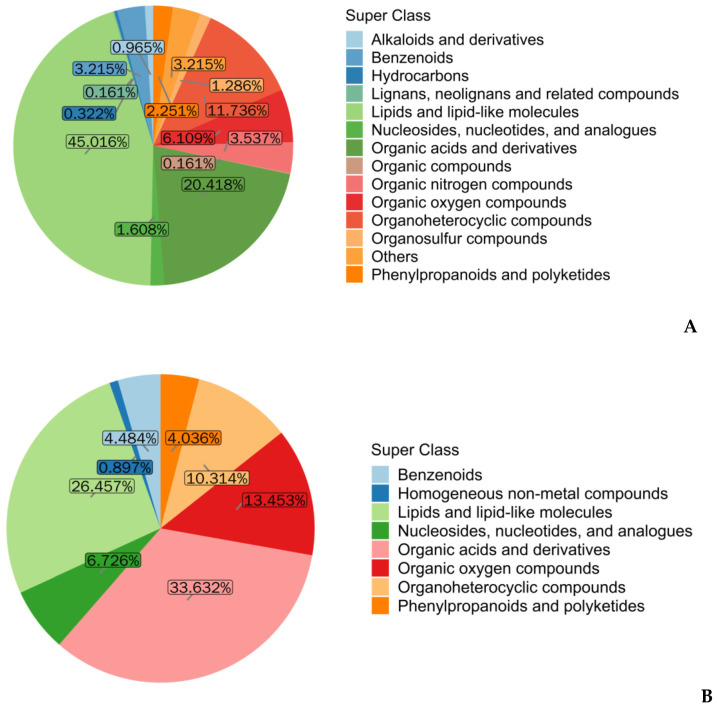
Classification of metabolites identified in boar sperm. (**A**) Major classes of sperm metabolites are identified in the positive ion mode. According to properties of metabolites, sperm metabolites identified in the positive ion mode were classified into different classes. The percentage of each class is shown in the pie chart. The colors represent each class of metabolite. (**B**) Major classes of sperm metabolites are identified in the negative ion mode. Sperm metabolites identified in the negative ion mode were classified into different classes. The percentage of each class is shown in the pie chart. The colors represent each class of metabolite.

In the positive ion mode, a total of 528 metabolites were identified in the sperm (data shown in the online repository). According to the chemical properties of the metabolites, the metabolites were classified into 16 categories. The main metabolites in the positive ion mode were lipids and lipid-like molecules, which contained 248 metabolites, accounting for 45.01% of all metabolites (Figure 3A) (Supplementary Table S1). The remaining metabolites were organic acids and derivatives (20.41%), organoheterocyclic compounds (11.74%), and the other 13 super classes (Figure 3A). In the negative ion mode, 194 metabolites in total were identified in the sperm (data shown in the online repository). The metabolites were classified into nine categories. Organic acids and derivatives were major metabolic classes and contained 61 metabolites, accounting for 33.63% of all metabolites (Figure 3B) (Supplementary Table S2). The remaining metabolites were separately distributed into the following classes: lipids and lipid-like molecules (51 metabolites, 26.45%), organic oxygen compounds (27 metabolites, 13.45%), and the other six classes (Figure 3B). Therefore, these data show that lipids and organic acids are the main metabolites in boar sperm.

### 3.4. Identification and Clustering Analysis of Differential Metabolites in Sperm

**Figure 4:**
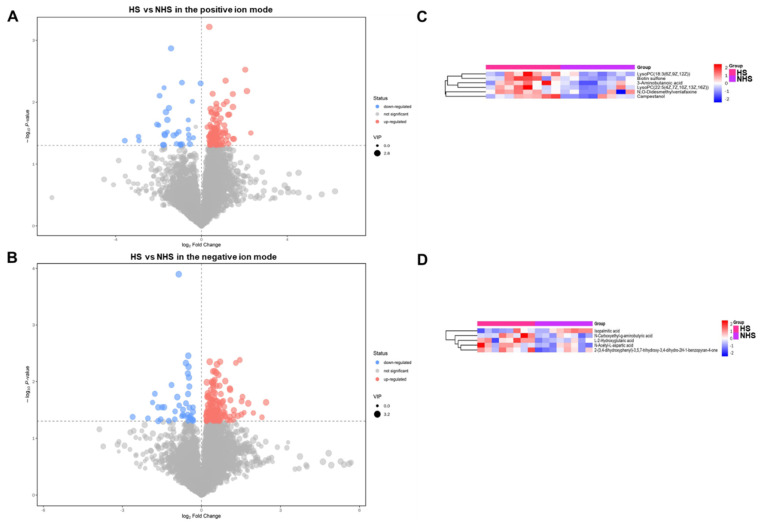
Differential expression and cluster analysis of metabolites between HS and NHS group. (**A**,**C**) Volcano plot of sperm metabolites identified in the positive and negative ion modes. The plots were used to display changes of metabolites between HS and NHS groups. The red circles represent up-regulated metabolites, the blue circles represent down-regulated metabolites, and the gray circles represent no changes of metabolites. (**B**,**D**) Heat map of sperm metabolites identified in the positive and negative ion modes. The heat maps were used to visualize the dynamic changes of metabolite content between HS and NHS groups. The pink represents HS group, and the purple represents NHS group. The color scale of the heatmap represents the gradual decrease of metabolite content from red to blue.

**Figure 5:**
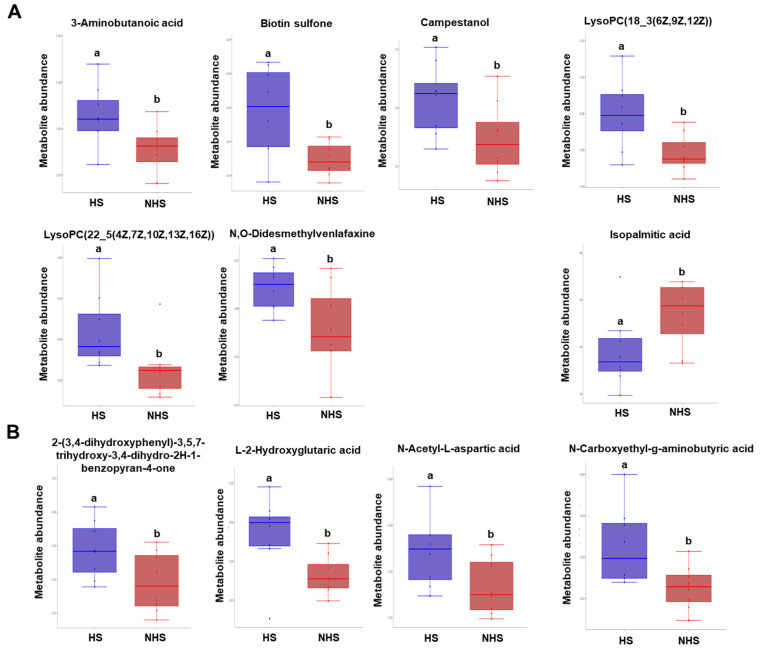
Verification of differential metabolites between HS and NHS groups. (**A**) Abundance of sperm metabolites in the positive ion mode between HS and NHS groups. The blue bars denote HS group, the red bars indicate NHS group. The metabolites identified in the positive ion mode included 3-aminobutanoic acid, biotin sulfone, campestanol, lysoPC(18_3(6Z,9Z,12Z)), lysoPC(22_5(4Z,7Z,10Z,13Z,16Z)), and N,O-Didesmethylvenlafaxine. The data are shown as mean ± S.E.M, and different letters on the bars indicate significant differences (*p* < 0.05). (**B**) Abundance of sperm metabolites in the negative ion mode between HS and NHS groups. The blue bars denote HS group, the red bars indicate NHS group. The metabolites identified in the negative ion mode included isopalmitic acid, 2-(3,4-dihydroxyphenyl)-3,5,7-trihydroxy-3,4-dihydro-2H-1-benzopyran-4-one, L-2-Hydroxyglutaric acid, N-Acetyl-L-aspartic acid, and N-Carboxyethyl-g-aminobutyric acid. The data are shown as mean ± S.E.M, and different letters on the bars indicate significant differences (*p* < 0.05).

The metabolites with variable importance in projection (VIP) > 1.0 and *p* < 0.05 were considered to be significantly changed. In the positive ion mode, we found that 163 metabolites in the HS group were significantly different from those in the NHS group, including 130 up-regulated and 33 down-regulated metabolites (Figure 4A). Among these, the chemical names for only six upregulated metabolites that contained 3-aminobutanoic acid, biotin sulfone, campestanol, LysoPC (18_3(6Z, 9Z, 12Z)), LysoPC (22_5(4Z, 7Z, 10Z, 13Z, 16Z)), and N, O-didesmethylvenlafaxine were determined. The heatmap of six differential metabolites is displayed in Figure 4B. As shown in the heatmap, samples in the same group clustered together, and the levels of each metabolite exhibited a differential change between the HS and NHS groups (Figure 4B). Furthermore, the differential expression of six metabolites was visible between HS and NHS groups (Figure 5A).

In the negative ion mode, we identified 171 differential metabolites in the HS group compared to the NHS group, including 131 up-regulated and 40 down-regulated metabolites (Figure 4C). Among them, the chemical names for only four up-regulated and one down-regulated metabolite, including isopalmitic acid, 2-(3,4-dihydroxyphenyl)-3,5,7-trihydroxy-3, 4-dihydro-2H-1-benzopyran-4-one, L-2-Hydroxyglutaric acid, N-Acetyl-L-aspartic acid, and N-Carboxyethyl-g-aminobutyric acid, were matched. The heatmap of five differential metabolites is displayed in Figure 4D. In addition, the quantitative analysis further revealed the differential expression of five metabolites between the HS and NHS groups (Figure 5B). Hence, these results demonstrate that certain metabolites exhibit differential expression between the HS and NHS groups.

### 3.5. Metabolic Pathway Analysis for Differential Metabolites in Sperm

**Figure 6:**
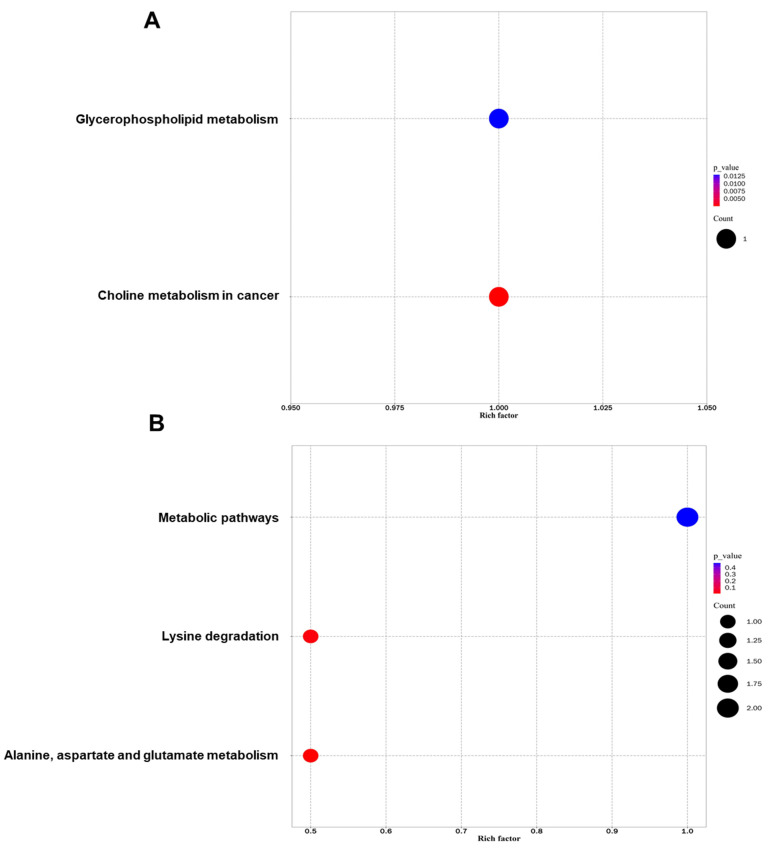
KEGG analysis of differential metabolites between HS and NHS groups. (**A**) Illustration of differential metabolite-enriched pathways in the positive ion mode. The color scale in the right of the bubble image represents gradual decrease of p-value from blue to red. Metabolic pathways are displayed in the left of the bubble image. (**B**) Illustration of differential metabolite-enriched pathways in the positive ion mode. The color scale in the right of the bubble image represents gradual decrease of *p*-value from blue to red. Metabolic pathways are displayed in the left of the bubble image.

All differential metabolites were subjected to metabolic pathway analysis using the KEGG database. In the positive ion mode, differential metabolites were mainly enriched in two significant metabolic pathways, involving choline metabolism and glycerophospholipid metabolism (Figure 6A). In the negative ion mode, differential metabolites were mainly involved in three significant metabolic pathways, including common metabolism pathways, alanine, aspartate and glutamate metabolism, and lysine metabolism (Figure 6B). In summary, differential metabolites were enriched in metabolic pathways important for sperm quality.

## 4. Discussion

HS significantly reduces semen quality and fertility in boars [4]. The ability of sperm to tolerate heat stress exhibits visible variations among individuals [5]. However, it is unclear if HS causes metabolic changes in swine sperm, and whether metabolic indicators for sperm thermal stress exist. In this study, we investigated the effects of HS on the kinetic features and metabolomic profiles of boar sperm, and further analyzed the potential functions of differential metabolites in sperm heat stress.

The kinematic parameters of sperm can not only be used to evaluate semen quality, but also can crudely predict male fertility [16]. We found that HS resulted in a significant reduction in sperm motility, APD, VSL, STR, and LIN, indicating the susceptibility of boar sperm to high temperatures. Consistent with our results, heat stress also altered the CASA parameters of sperm in boars [6] and buffalo bulls [15]. Unexpectedly, HS only reduced the percentage of viable sperm, but did not harm sperm motility and other motion parameters in rabbits [17]. This discrepancy in sperm motion characteristics might be due to differences between species.

Metabolites are classified based on their composition as amino acids, lipids, carbohydrates, nucleotides, minerals, and vitamins. We found in the positive and negative ion mode that the proportion of lipid molecules and organic acids was maximal in native boar sperm, respectively. Similarly, organic acids were major metabolic classes in the seminal plasma of commercial boars [18]. A previous study showed that the major metabolic class in bull sperm was also organic acids [19], indicating that organic acids might have important functions in the sperm of different species.

In addition, we identified that heat-stressed sperm generated 160 or 171 differential metabolites in the two ion modes. Studies have shown that differential metabolites related to sperm quality mainly involved lysophosphatidylcholine [20], palmitic acid [21], and γ-aminobutyric acid [22]. Specifically, lysophosphatidylcholine supplementation reportedly induced an acrosome reaction of sperm in rabbits [23] and bulls [24]. Palmitic acid is the most abundant saturated fatty acid in boar sperm plasma membranes [25], and could improve boar sperm motility via mitochondrial B-oxidation [21]. γ-aminobutyric acid addition enhanced the capacitation and acrosome reaction of sperm in several species [26,27,28,29]. Given the association between the above-mentioned differential metabolites and sperm functionality, it is conceivable that these metabolites could be regarded as potential biomarkers of heat stress for boar sperm.

Differential metabolites identified in this study are mainly enriched in the metabolic pathways of glycerophospholipid, choline, and amino acids. These metabolic pathways participate in the regulation of sperm quality. For instance, glycerophospholipid incorporated into sperm membranes can reduce oxidative damage and improve stallion sperm quality [30]. Glycerophospholipid supplementation significantly alleviated oxidative stress, and enhanced the motility and viability of fresh and post-thaw sperm in humans [31,32]. Dietary choline was required for sperm motility in Drosophila [33]. Lysine acetylation was involved in regulating boar sperm motility and acrosome integrity [34], and inducing a mouse sperm acrosome reaction [35]. Alanine addition improved the post-thaw motility of striped bass sperm [36]. D-aspartic acid supplementation elevated the quality of rooster post-thaw sperm [37], rabbit buck fresh sperm [38], and the fertilizing capacity in mice [39]. Thus, it may be possible to developing tools to mitigate the heat stress of boar sperm via modulation of these metabolic pathways.

Numerous studies indicated that HS caused a significant reduction in human sperm quality [40,41,42]. Differential metabolites and metabolic pathways identified in this study might be relevant to human sperm quality. For example, mRNA levels of γ-aminobutyric acid receptors in human sperm are correlated with poor sperm quality [43]. D-aspartate could protect human sperm via preventing the decrease of motility and the increase of DNA fragmentation and lipid peroxidation [44]. Moreover, lysine acetylation and glutarylation in human sperm were well associated with sperm functions [45,46]. Thus, these results could provide a useful reference for identifying the HS biomarkers of human sperm.

## 5. Conclusions

Our results demonstrate that HS alters the motion characteristics and metabolomic profiles of boar sperm. These findings have implications for screening heat-tolerant animal and human semen, and for developing strategies to mitigate HS.

## Data Availability

The raw data presented in the study are deposited in the MetaboLights repository (accession number: MTBLS5391).

## Supplementary Materials

The following supporting information can be downloaded at: https://www.mdpi.com/article/10.3390/genes13091647/s1, Table S1: The percentage of major classes of sperm metabolites identified in the positive ion mode; Table S2: The percentage of major classes of sperm metabolites identified in the negative ion mode.
